# Mapping the relationship and influence of school internal factors with an eye towards students' science academic outcomes

**DOI:** 10.1016/j.heliyon.2024.e38696

**Published:** 2024-09-27

**Authors:** Hafiz Muhammad Ihsan Zafeer, Samra Maqbool, Yu Rong, Sufyan Maqbool

**Affiliations:** College of Education, Zhejiang Normal University, Jinhua, Zhejiang, 321004, China

**Keywords:** School internal factors, Science academic outcome, Secondary schools, Pakistan

## Abstract

The present research sought to explore the relationship between school internal factors and their influence on students' academic outcomes in science subjects at the secondary school level in Punjab, Pakistan. A quantitative survey method was employed, utilizing a self-administered questionnaire to collect data on school internal factors, including laboratories, curriculum, and teacher quality, and students' science academic outcomes. The study sampled 210 secondary schools across Punjab, encompassing 630 science teachers specializing in biology, physics, and chemistry. Three hypotheses were formulated and tested. The constructs' underlying structure was examined through both exploratory and confirmatory factor analyses, using the PLS-PM Path Modeling approach to support these analyses. Structural Equation Modeling (SEM) revealed that the measurement model demonstrated internal consistency. The path analysis results indicated that laboratories, curriculum, and teacher quality significantly and positively influence students' academic outcome in science subjects. The study concludes that enhancing the resourcefulness of science educators is crucial, particularly through the provision of support materials for science teaching and learning. This support enables students to engage in experimental learning, develop critical thinking skills, and explore innovative approaches to problem-solving. Furthermore, the findings emphasize the importance of regularly reviewing and updating the secondary school curriculum to ensure its quality. The recruitment of certified teachers with advanced degrees in relevant fields and the provision of ongoing professional development for educators are also recommended to improve academic outcomes in science.

## 1 Introduction

The relevance of science in a country's education system has expanded due to its usefulness and significance. All nations' educational systems have emphasized science education. Most nations teach science in elementary school [1]. It allows students to gain knowledge and understanding of the fundamental principles and concepts in physics, chemistry, biology, and mathematics, which together include the many aspects of the natural world [2]. It promotes the cultivation of appropriate scientific attitudes among learners and enhances their capacity for future academic pursuits. The process of fostering scientific knowledge, attitudes, and skills should not rely only on transferring cultural norms and practices from generation to generation. Instead, it should prioritize actively building ideas, knowledge, scientific attitudes, skills, and competencies via a practical and intellectually engaging approach [3].

Academic performance is defined by Wang and Degol [4], as the outcomes and accomplishments that students achieve while studying in a classroom. These performances can be positively or negatively demonstrated due to various factors, including the availability or non-availability of school experimental laboratories, curriculum, and teacher quality. The explanations of current study's variables has been elaborated in Table 1.

**Table 1:** Current research variables and their explanation.

Variables	Explanation
Science Laboratories	Laboratory facilities are designed for scientific experimentation, research, and teaching. These labs allow scientists, researchers, and students to study physics, chemistry, biology, geology, and environmental science in a controlled setting.
Curriculum	A curriculum is an organized strategy to help pupils meet learning goals. It includes subject-specific material, instructional techniques, assessments, and educational experiences.
Teacher Quality	A teacher's knowledge, abilities, and dispositions that contribute to successful teaching and favorable student outcomes make up teacher quality.

The term “doing science” is referred to by various names in science education research. These include experiments, science inquiry, laboratory work, hands-on work, and science practices [5]. As indicated by Akçayır, Akçayır [6] a scientific laboratory is an educational resource that teaches students about science and the methods used by scientists. It offers a platform for students to apply the theoretical information acquired inside the confines of the classroom. This application enhances their comprehension and facilitates the integration of theoretical knowledge with practical application. Moreover, incorporating technology within experimental configurations may significantly augment the educational encounter for students [7]. Providing well-equipped experimental laboratories inside educational institutions can positively impact students' academic performance, particularly in science, technology, engineering, and mathematics (STEM).

The *curriculum* is an influential component that significantly impacts students' academic outcomes, including their success in scientific disciplines. A science curriculum that is thoughtfully created and implemented may play a significant role in enhancing students' comprehension of scientific principles, cultivating their ability to think critically, and nurturing a sustained enthusiasm for the discipline [8]. According to scholars, curriculum affects students' science academic achievement in numerous ways, as shown in Table 2.

**Table 2:** Affective ways of curriculum and their description.

Affective Ways	Description
Relevancy Content	A curriculum that relates to students' lives might motivate them. Applying scientific principles to real-world situations helps students understand their relevance [9].
Scope	The scope and chronology of a scientific curriculum play a crucial role in shaping the evolution of subjects and ideas that students encounter throughout their academic trajectory—implementing a meticulously designed curriculum guarantees that students progressively develop their core knowledge and abilities, fostering a holistic comprehension of the field of science [10].
Inquiry-Based Learning	Curricula that include practical experiments and inquiry-based learning methodologies give students tangible opportunities for experiential learning. These strategies facilitate comprehension and cultivate curiosity and a passion for scientific inquiry [11].
Interdisciplinary Association	Incorporating scientific principles into several fields, including mathematics, technology, and the arts, may provide a comprehensive and integrated perspective on the world. An interdisciplinary approach could augment students' capacity to apply scientific concepts across various circumstances [12].
Teacher Professional Development	A scientific curriculum's effectiveness relies on instructors' preparation and skill. Professional development helps teachers use the newest instructional techniques and technology to deliver the curriculum [13].
Promoting Scientific Inquiry Skills	A good science curriculum emphasizes observation, questioning, experimentation, and analysis. These qualities are crucial for academic and professional scientific achievement [14].

Similarly, an analysis of the empirical research has unveiled an ongoing discourse regarding the impact of teacher qualities on student achievement. Over the past decade, research has established that many factors influence student achievement; however, teacher quality has emerged as the most remarkable determinant [15]. Moreover, effective pedagogical strategies, such as hands-on experiments, demonstrations, and real-world applications, can potentially augment students' levels of engagement and understanding of scientific ideas. Diverse teaching methods accommodate varied learning styles, creating a more inclusive and helpful learning environment [16].

The focus on teacher effectiveness and student achievement has intensified due to the implementation of new learning standards in several jurisdictions [17]. The link between the quality of teachers and the academic success of students in the field of science is a subject that is intricate and encompasses several dimensions [18]. Despite differing findings, several scientific studies have shown that teacher quality strongly affects student academic performance [19]. Educators with a robust understanding of scientific subject matter are more adept at effectively communicating complex ideas and fostering engaging discourse among learners. Teachers can answer students' queries and explain scientific ideas better if they understand the subject [20].

However, developing nations have millions of opportunities for scientific education development while many emerging nations are attempting to develop their scientific education. Although scientific education in Pakistan is poor, it could be better, especially at the school and college levels. The insufficiency of scientific facilities in secondary schools across Pakistan poses a substantial and complex obstacle, hampering the caliber of science instruction and obstructing the academic growth of learners. A significant number of secondary schools in Pakistan are deficient in well-equipped scientific labs, which are crucial for facilitating hands-on experimentation and the practical application of theoretical notions. The lack of contemporary laboratory facilities impedes the development of critical laboratory skills among students and hinders the attainment of a comprehensive understanding of scientific principles. Practical teachings are hampered by a lack of current and functioning scientific curricula. Outdated or inadequate resources limit the breadth of experiments, preventing students from grasping complicated scientific processes. Further, educators often have difficulties in efficiently using existing scientific resources as a result of insufficient training in integrating practical components into their instructional approaches. The limited availability of professional development opportunities and training programs for educators hinders the potential effectiveness of the existing resources, hence influencing the academic outcomes of students.

Thus, the present study aimed to explore/determine school internal factors that influence students' science academic outcomes, understand the influence of school internal factors on students' science academic outcomes and determine the relationship between school internal factors and students' science academic outcomes. Based on above mentioned aims following research questions were formulated; 1) Do school internal factors like; Laboratories, Curriculum and Teacher Quality influence students' science academic outcomes at the secondary level? 2) What are the relationship of school internal factors like; Laboratories, Curriculum and Teacher Quality influence with students’ science academic outcomes at secondary school level?

## 2 Theoretical underpinning

The research was influenced with constructivist theory of learning. Constructivism is an educational approach that prioritizes experiential (science) learning and the active engagement of students in their own learning process [21]. Three strategies stand out in this context: project-based learning, the discovery approach, and the conceptual transformation method [22]. John Dewey was the first to promote the concept of hands-on activities and experiential learning. This theory challenged the conventional belief that learning occurs only via lectures and rote memorization [23]. The notion of experiential learning highlights the significance of active physical participation in the process of acquiring knowledge. Science labs should include hands-on activities and experiments that enable students to handle materials, make observations, and conclude, therefore reinforcing theoretical ideas via direct experience [24]. According to the instructional theory of learning interaction, the scientific laboratory significantly impacts students' attitudes and academic accomplishments [25]. Cognitive apprenticeship theory posits that learning is a collaborative process in which individuals who lack expertise acquire knowledge and skills from those with advanced knowledge. It promotes the active participation and cooperation of students and instructors [26,27].

Lev Vygotsky's Zone of Proximal Development (ZPD) theory is applicable in secondary schools, suggesting that students get advantages by engaging in more challenging activities than their present level of ability [[28], [29], [30]]. Further, essentialism theory prioritizes the imparting of fundamental information and skills to students. Within science education, an essentialist curriculum emphasizes the key scientific ideas and principles that are considered vital for every learner [31,32]. In addition, pedagogical content knowledge (PCK) theory refers to the particular expertise that instructors must possess to instruct specific subject matter proficiently. In science education, educators with robust Pedagogical Content Knowledge (PCK) can effectively convey scientific ideas to students, predict and address frequent misunderstandings, and create impactful teaching methods [19,[33], [34], [35], [36]]. However, constructivist teaching theories also emphasize the teacher's role as a facilitator of learning. Effective science educators promote student inquiry, discovery, and hands-on experiences, enabling students to create their comprehension of scientific topics via active participation [37,38].

## 3 Literature review and construction of hypothesis

### 3.1 The role of science laboratories in students' science learning

The science laboratory plays a vital role in our efforts to diversify the learning environment in which students enhance their comprehension of scientific topics, science inquiry skills, and perspectives of science [39,40]. It is a distinctive educational space where students may collaborate in small groups to explore scientific phenomena [41]. However, learning scientific knowledge helps students develop practical methods and procedural skills, understand fundamental scientific principles, concepts, terminology, and facts, apply scientific knowledge in everyday life, and become aware of current scientific issues and advancements [42]. Further, science education greatly benefits from access to laboratories. In addition to the core subjects of physics, biology, and chemistry, laboratory lessons are an integral aspect of a well-rounded scientific education. In order to grasp theoretical ideas, laboratory work is necessary [43]. Şahin Pekmez, Aktamış [44] questioned why science instructors needed to conduct classroom experiments. Teachers suggested helping students understand and learn better, increasing their interest in classes, improving their manual skills, helping them discover knowledge independently, improving their observation and problem-solving skills, and ensuring they learn through experience. Lab experiments offer numerous benefits, but integrating them with other teaching methods may maximize their effectiveness and provide students with a complete education [45]. Moreover, well-equipped labs and proficient teachers may significantly influence the effectiveness of laboratory-based educational endeavors. Hence, we hypothesized.

H1 There is positive influence of science laboratories on students' science academic outcomes at secondary school level.

### 3.2 The role of scientific curriculum and students’ academic outcomes

The successful instruction and acquisition of scientific knowledge in high school mainly rely on the accessibility of curricular material for both teachers and learners [46]. Shawer [47] and Pullin [48] define curricular content as the collection of laws, principles, ideas, theories, facts, and generalizations that constitute the subject matter of different disciplines. Curriculum content refers to the comprehensive knowledge and subject matter that is imparted and acquired inside an educational institution [49]. The adage “knowledge is power” suggests that curricular material is the primary source of knowledge, skills, and attitudes gained in school since information is the foundation of knowledge [50]. However, students' access to curricular information may impact their learning and, ultimately, their academic achievement. Akande and Bamise [51] contend that the origin and availability of information might impact the scholastic achievement of secondary school learners. Biology, Chemistry, and Physics are crucial since they create the fundamental knowledge base taught and learned in school. Proficiency in biology is essential for establishing a solid foundation for prospective careers in medicine, agriculture, nutrition, and related fields [52].

As previously stated, scholars believe that the development and availability of curriculum, material, and information may influence students' academic achievements. Curriculum is not limited to textbooks; it can be accessed through the library and the Internet. The school library is a crucial component of any educational system, providing assistance and advice to readers [53]. Chao, Chen [54] stated that the school library needs to have enough resources and be easily accessible to students and instructors. A well-curated library collection is an essential resource that improves the quality of education and promotes high academic standards. The internet is another significant resource that students may use to get or retrieve subject matter knowledge. As indicated by Yang, Balaji [55] students' academic progress has been greatly aided by the availability of up-to-date, easily searchable material on the internet. The Internet helps students expand their academic knowledge, research, and complete assignments by providing access to material from across the globe [56].

Furthermore, scholars contend that it is advantageous to regularly update and revise the science curriculum to keep pace with the ever-evolving nature of the field, such as *Continuous Advancements in Scientific knowledge:* by updating the curriculum, students can acquire information about the most recent scientific discoveries, guaranteeing that their understanding remains up-to-date and applicable [57]. *(2) Technological Changes:* The instruments and strategies used in scientific investigation undergo progressive changes throughout time. An updated curriculum may include emerging technology, offering students practical involvement and equipping them with the necessary skills for contemporary scientific methodologies [58]. *(3) Critical Thinking and Problem-Solving:* An effective curriculum should prioritize the development of critical thinking and problem-solving abilities. Regular updates may include innovative teaching techniques and approaches that improve these abilities, thus better equipping students to tackle future difficulties [59]. *(4) Relevance to Real-World Issues:* Science is essential in tackling global climate change, public health emergencies, and technology advancements. A revised curriculum may more effectively correspond with real-world concerns, enhancing students' comprehension of the pragmatic implications of scientific knowledge [60]. Thus, we assumed that.

H2 There is positive influence of curriculum on students' science academic outcomes at secondary school level.

### 3.3 The role of teacher quality and students’ academic outcomes

After family background, teacher quality is the second most crucial element in student success. Students with good teachers learn more than those with poor ones, and their academic outcomes determine teacher quality [61]. An analysis of the empirical research has shown an ongoing discussion over the impact of teacher qualities on student achievement. Bruns and Luque [62] argued that student variables are the most influential predictors of student accomplishment. Maamin, Maat [63] conducted a comprehensive literature analysis and found that teacher quality is the most influential factor affecting student success. However, Goe [64] devised a framework that enhances comprehension of teacher quality. The framework identified and characterized essential components of teacher quality and demonstrated the correlation between these components and student learning. Teacher quality within the framework is determined by teacher training, education, certification, credentials, and experience. These factors together affect the quality of classroom teaching, which in turn influences student learning results [65]. In addition, Blömeke, Olsen [20] regarded teacher credentials, educational background, teaching experience, personality qualities, and professional growth as essential factors in determining teacher quality. Similarly, Kola and Sunday (2015) examined the quality of teachers in Nigeria by assessing seven factors, such as credentials and training. Their research revealed that students' academic success is influenced and favorably associated with their subject matter knowledge, pedagogy studies, professional growth, and years of experience [66].

Moreover, Prior studies on teacher quality have critically focused on assessing the impact of teachers on student performance in subjects such as science or mathematics [67,68]. While a considerable amount of research shows teacher quality's influence on student accomplishment, there still needs to be an agreement on which specific elements of teacher quality have the most significant effect on student performance [69,70].

Thus, teachers with scientific competence may help students learn meaningfully, and a thorough understanding of scientific concepts allows teachers to explain complicated topics, answer questions, and educate students [71]. Likewise, teaching science requires a comprehensive grasp of the topic and suitable teaching methods. Trained teachers can develop engaging classes, use a range of instructional approaches, and customize their teaching methods to their students' requirements, which improves learning outcomes [72,73]. Therefore, we expect that.

H3 There is positive influence of teacher quality on students' science academic outcomes at secondary school level.

**Fig. 1:**
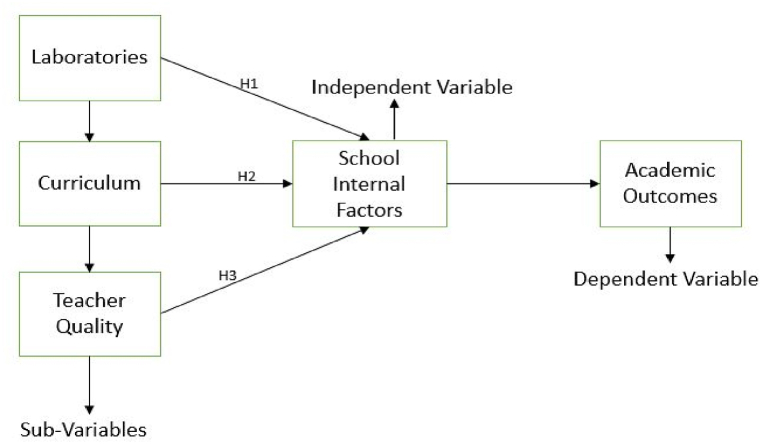
Study conceptualization and hypothesis.

Based on research questions theories and discussed literature, it was postulated that internal variables inside schools impacted students' academic outcomes in science. The internal factors of the school are further categorized into three sub-variables, i.e., laboratories, curriculum, and teacher quality (independent variables). The students' science academic outcomes was considered as a dependent variable are illustrated in Fig. 1.

## 4 Materials and method

### 4.1 Design and participants

The current research used the quantitative survey approach [74,75]. The participants were selected using convenient sampling from 210 secondary schools located in Punjab, Pakistan. The participant sample consisted of 630 secondary school teachers presently instructing science courses such as Biology, Physics, and Chemistry. Prior to data collection, informed consent was acquired from each participant.

### 4.2 Research instruments

The survey questionnaire was developed for data collection. The questionnaire was developed using substantial educational literature and existing research on the subject matter like; [[76], [77], [78], [79], [80], [81]]. It was then updated to align with the research questions and hypothesis. We designed the questionnaire to assess school internal factors, dividing it into four variables. We included three independent variables, such as laboratories (consisting of 5 items), curriculum (consisting of 6 items), teacher quality (consisting of 5 items), and students’ science academic outcomes (consisting of 5 items), as one of the dependent variables. The data of independent variables were collected through a five-point Likert scale, with 1 indicating “strongly disagree” and 5 indicating “strongly agree”. The data of the dependent variable (academic outcomes) was collected using a five-point Likert scale, with 1 indicating “poor” and 5 indicating “excellent.” The complete survey questionnaire has been attached in a supplementary file.

### 4.3 Validity, reliability and pilot study

The first survey instrument/questionnaire had a total of 25 items. Three highly competent experts in this domain examined the instruments/questionnaire to verify the questionnaire's face validity. Four items were excluded after the implementation and expert opinions, resulting in a final formulation of the survey instruments/questionnaire comprising 21 items. The questionnaire consists of four variables, including three independent variables: laboratories, curriculum and teacher quality, and one dependent variable, students' science academic outcomes. Cronbach's alpha was computed to assess the reliability of the survey instruments/questionnaire. For this, a questionnaire pilot study was carried out with a sample of 186 individuals. The alpha coefficient indicated a strong level of internal consistency with the value of (α = 0.89), indicating that the questionnaire is very trustworthy for collecting formal data, as supported by the literature review [82,83]. The Cronbach's Alpha scores for each variable are shown in Table 5.

### 4.4 Data collection and analysis procedure

Participants were contacted at their schools on prearranged days with their agreement to collect data. The survey sheets were distributed to the participants, accompanied by a consent form, to get their authorization to participate in this study. Furthermore, it was explicitly explained to them that their information would be treated with the utmost confidentiality and only used for educational purposes, ensuring their identity remained anonymous. Participants are free to withdraw from the survey at any moment if they choose not to participate. Six hundred fifty (650) sheets were given, of which 639 were returned. Nine (9) sheets were omitted due to incomplete answers and missing data. Before undertaking analysis, the Kolmogorov-Smirnov test was used to assess the normal distribution of the data [84]. Based on the findings shown in Table 3, the data exhibited a normal distribution and is appropriate for additional analysis [85]. Moreover, the data was analyzed using the Partial Least Squares (PLS-PM) Path Modeling technique, as described in previous studies [[86], [87], [88]]. This approach was used to assess the influence and relationship between independent and dependent factors.

**Table 3:** Results of the Kolmogorov–Smirnov test.

Variables	Codes	Kolmogorov–Smirnov	Asymp. Sig.(2-tailed)
Laboratories	Labs	0.105	0.000
Curriculum	CM	0.102	0.000
Teacher Quality	TQ	0.127	0.000
Academic Outcomes	AO	0.083	0.000

## 5 Findings

Table 4 presents the demographic characteristics of the sample, categorized into six variables: age, gender, academic qualifications, professional qualifications, experience, and teaching course. These variables were analyzed using frequency, percentage, mean, and standard deviation. The results are as follows: age (M = 1.60, SD = 0.575), gender (M = 1.33, SD = 0.472), academic qualifications (M = 1.66, SD = 0.529), professional qualifications (M = 1.31, SD = 0.625), teaching course (M = 2.03, SD = 0.737), and experience (M = 1.17, SD = 0.383).

**Table 4:** Demographic Characteristics for participants.

Variables	F	%	M	SD
*Age*				
25–35	277	44.0	1.60	0.575
36–45	324	51.4		
Above 46	29	4.6		
Total	630	100.0		
*Gender*				
Male	419	66.5	1.33	0.472
Female	211	33.5		
Total	630	100.0		
*AQ*				
B.A/B.Sc	330	36.5	1.66	0.529
M.A/M.Sc	282	60.6		
M.phill	18	2.9		
Total	630	100.0		
*PQ*				
B.Ed	486	77.1	1.31	0.625
M.Ed	89	14.1		
Don't Have	55	8.8		
Total	630	100.0		
*TS*				
Biology	159	25.2	2.03	0.737
Physics	287	45.6		
Chemistry	184	29.2		
Total	630	100.0		
*Experience*				
1–10	517	82.1	1.17	0.383
11–20	113	17.9		
Total	630	100.0		

Note: F = frequency, % = percentage, M = mean, SD = standard deviation, AQ = academic qualifications, PQ = professional qualifications, TS = teaching subjects.

## 6 Assessment of the measurement model

**Fig. 2:**
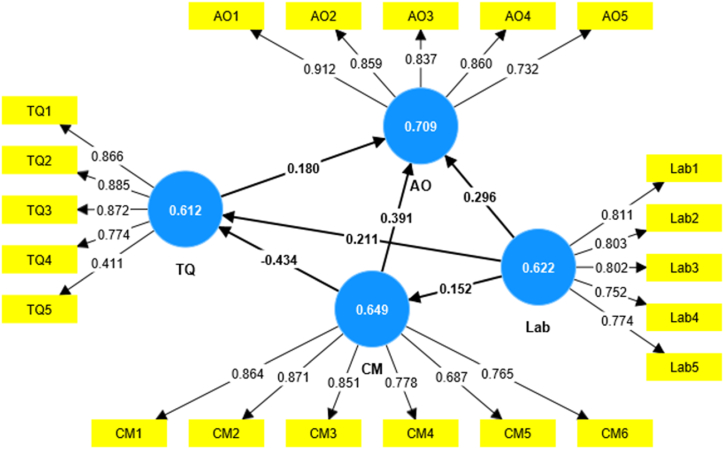
Measurement path analysis model.

Confirmatory factor analysis (CFA) is used to verify that the measurement model is accurate. According to scholars evaluating the measurement model may be done by checking for multicollinearity, discriminant, and convergent validity [[89], [90], [91], [92]]. The convergent validity was assessed by examining the extracted values of factor loadings, composite reliability, and average variance. The measurement model shown in Fig. 2 was generated by executing the PLS algorithm. Factor loadings greater than 0.7 are required for convergent validity, as stated by Refs. [90,93] Table 5 displays the results of the CFA factor loadings. Except CM5 and TQ5, with loadings of 0.687 and 0.511, respectively, were below the 0.7 suggested threshold by Ref. [90] in Table 6, which shows that the other outer loadings (CFA) were too high. Nevertheless, these items were included because of their content validity and because removing them did not enhance the AVE values further [90]. The researchers believed composite reliability should exceed 0.7 [90]. According to Table 5, all constructs surpassed the 0.7 criteria (ranging from 0.882 to 0.924), demonstrating that the model has achieved reliability. Additionally, the Average Variance Extracted must exceed 0.5. Table 5 shows all the findings above the criterion of 0.5, with values ranging from 0.612 to 0.709 [90]. Table 5 displays the results of the figure for measurement model indices, which demonstrate that the measurement model achieved internal consistency.

**Table 5:** Metrics for internal consistency in structural model.

Factors	Indicators	Loadings (λ)	VIF	CR	Alpha(α)	AVE
Laboratories	Lab1	0.811	2.020	0.892	0.848	0.622
	Lab2	0.803	1.948			
	Lab3	0.802	1.973			
	Lab4	0.752	1.621			
	Lab5	0.774	1.660			
Curriculum	CM1	0.864	2.848	0.917	0.891	0.649
	CM2	0.871	2.791			
	CM3	0.851	2.690			
	CM4	0.778	2.048			
	CM5	0.687	1.684			
	CM6	0.765	1.801			
Teacher Quality	TQ1	0.866	2.611	0.882	0.830	0.612
	TQ2	0.885	2.926			
	TQ3	0.872	2.521			
	TQ4	0.774	1.596			
	TQ5	0.511	1.136			
Academic Outcome	AO1	0.912	2.583	0.924	0.896	0.709
	AO2	0.859	2.774			
	AO3	0.837	2.442			
	AO4	0.860	2.826			
	AO5	0.732	1.748

**Table 6:** Discriminant validity through Heterotrait-monotrait ratio (HTMT) rules.

Factors	Labs	CM	TQ	AO
Lab	–			
CM	0.390	–		
TQ	0.434	0.174	–	
AO	0.083	0.446	0.176	–

Note: Labs = laboratories, CM = curriculum, TQ = teacher quality, AO = academic outcomes.

The differentiation between the constructs was established using discriminant validity [94,95]. This research used the HTMT (Heterotrait-Monotrait Ratio) metric. Henseler, Ringle [96] and Dirgiatmo [97] state that the Heterotrait-Monotrait Ratio should not exceed 0.85 (strictly) or 0.90 (as an acceptable value). All of the constructions were distinct, as seen in Table 6. Once the validity and reliability of the measurement model have been confirmed, the subsequent step involves constructing the structural model, examining the various paths, and evaluating the hypotheses.

## 7 Description of path analysis and hypothesis testing

**Fig. 3:**
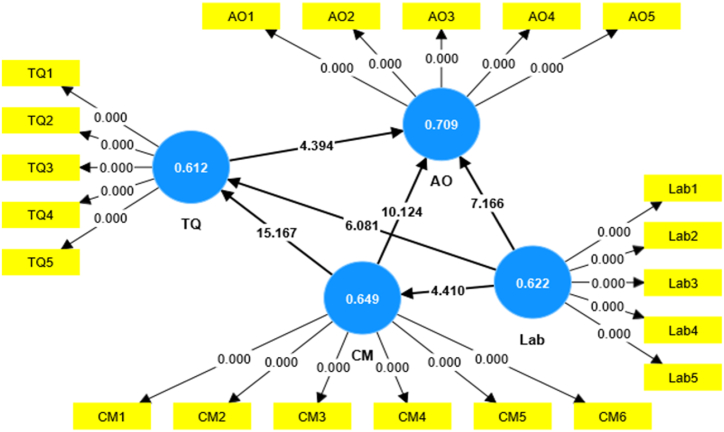
Bootstrap model for path analysis.

After assessing the measurement model's convergent and discriminant validity, the t values were determined using the PLS bootstrapping (BT) approach to analyze the relationships between latent variables. Fig. 3 displays the path coefficients and t-values the arrows represent, indicating whether they are significant or non-significant relationships. The requirement is for the t-values to be larger than 1.96 and have a p-value less than 0.05. The findings in Table 7 and Fig. 3 predicts that H_1_ there is positive influence of science laboratories on students' science academic outcomesat secondary school level with (t = 7.166, β = 041, p < 0.000∗∗∗), H_2_: there is positive influence of curriculum on students' science academic outcomes at secondary school level with (t = 10.124, β = 039, p < 0.000∗∗∗) and H_3_: there is positive influence of teacher quality on students' science academic outcomes at secondary school level with (t = 4.394, β = 044, p < 0.000∗∗∗) were significant influence and relationship students' science academic outcomes.

**Table 7:** Hypothesis testing using path analysis.

Relationship	Path Coefficients	Β	T	P	f^2^	2.5 %	97.5 %	Decision
Lab → AO	0.296	0.041	7.166	0.000	0.111	0.216	0.377	Accepted
CM → AO	0.391	0.039	10.124	0.000	0.165	0.315	0.465	Accepted
TQ → AO	0.180	0.044	4.394	0.000	0.035	0.099	0.259	Accepted

Note: β = beta value, T = t-statistics, P = p-values, f^2=^f-square.

The Goodness-of-Fit (GOF) measure is used to assess a model's overall fit and determine whether it adequately clarifies the observed data [98,99]. In terms of model fit, the model underwent many iterations before achieving a satisfactory fit. The CFA model indicated earlier (refer to Fig. 3) was determined to fit the data adequately after several iterations, as suggested by researchers [86,100]. The goodness of fit indices of the final CFA model are shown in Table 8, indicating that a satisfactory model fit has been achieved.

**Table 8:** Goodness of fit indices for measurement model.

Fit Indices	Obtained Value
SRMR	0.059
Chi-square *(X*^*2)*^	1077.794
P-Value	0.000
NFI	0.859

Note: SRMR = standardized root mean square residual, X^2^ = chi-square, NFI = Normed Fit Index.

## 8 Discussion

Science education provides students with the necessary abilities to succeed in various professions, ensuring they are ready for future challenges and possibilities. In developing countries, there has not been much research on how school internal factors influence students' academic outcomes in science courses at school, college, and university levels. Pakistan is one of a developing nations there also needs to focus on the science education sector, especially at the secondary school level. The present study aimed to determine the school's internal factors like laboratories, curriculum, and teacher quality that influence students' science academic outcomes. The results indicated that science labs, curriculum, and teacher quality influenced students' science academic outcomes.

Within the realm of education, science education encompasses the pedagogical activities involved in imparting and acquiring knowledge about scientific content, ideas, structures, facts, hypotheses, theories, scientific laws, procedures (approaches), attitudes, and scientific abilities [101,102]. It enhances the education of future scientists and promotes a broader and more relevant awareness of nature and scientific results among the general people [103]. It provides students with an understanding of technology and cultivates their own experiences. Encouragement of practical skills in technology activities helps youngsters gain knowledge, resources, and mental and physical abilities [104]. However, integrated science is a multidisciplinary field that encompassing several scientific disciplines, including Biology, Microbiology, Ecology, Physics, Chemistry, Earth Science, and Astronomy [105]. UNESCO has identified many justifications for implementing integrated science as a component of fundamental education across diverse nations. Several causes may be identified: (1) incorporating integrated scientific education at primary and secondary levels may provide a solid foundation for students to further their understanding of integrated science or specialized disciplines. (2) The advancement of contemporary scientific inquiry has resulted in the emergence of an interdisciplinary approach within the field of science [106]. According to Nadelson, Heddy [107] integrated science is a subject that seeks to amalgamate thoughts, perspectives, and methodologies from several scientific fields to elucidate scientific phenomena seen in daily existence.

Moreover, science labs are a strong predictor pivotal in augmenting the educational experience and comprehension of scientific principles, ultimately leading to students' academic success [108]. The absence of scientific labs in schools limits students' hands-on experiences, hinders their development of important skills, and lowers their science academic performance [77]. However, well-equipped scientific labs are necessary for a complete science education [109,110]. In scientific education, Hofstein and Mamlok-Naaman [111] suggested that lab work helps students understand data observation vs. data presentation. Similarly, Laboratory activities allow students to comprehend and create knowledge via science [112,113]. Likewise, the previous researcher found a link between hands-on activities and academic achievement, showing that practical experiences improved scientific knowledge and performance [114,115].

In addition, by analyzing several studies, Hofstein and Lunetta [116] also examined how lab experiences affect science students' academic achievement. The findings revealed that laboratory activities improved science academic achievement, and the result was greater when lab activities were included in the scientific curriculum. However, earlier research suggested that curriculum design and execution affect students' scientific classroom achievement [117]. The correlation between curriculum and student achievement is an intricate and diverse subject, and educational research has examined several facets of this association [118]. Martone and Sireci [119] stated that implementing a demanding and stimulating curriculum that aligns with students' cognitive abilities may increase academic performance. This study's findings align with the findings of Karvonen, Wakeman [120], who demonstrate that students' interest and achievement in science are higher in curricula that include experiential and experimental learning opportunities. Conversely, research emphasizes the beneficial effects of incorporating technology into scientific curricula. Utilizing virtual laboratories, simulations, and interactive multimedia materials may enhance students' comprehension and achievement in science [121].

Meanwhile, research on teacher quality and student outcomes is an area of interest for educational researchers [122]. Teacher quality encompasses a teacher's total efficacy, proficiency, and characteristics within education [123,124]. The relationship implies that improving students' favorable disposition towards science might facilitate their acquisition of scientific knowledge. Savelsbergh, Prins [125] conducted a quantitative literature synthesis demonstrating the potential of context-based teaching methods, such as inquiry-based learning, technology-based learning environments, collaborative learning, and extracurricular activities, to greatly improve students' overall attitude towards science. The meta-analytic study conducted by Aguilera and Perales-Palacios [126] revealed that certain teaching methods and approaches, such as cooperative learning, project-based instruction, context-based instruction, and the use of technology-multimedia materials, have the potential to influence students' science academic outcomes positively. In the future, scientific education may use learning methodologies and innovative teaching approaches, such as flipped learning and game-based learning, to foster a more favorable attitude toward science among students.

## 9 Limitations and future research directions

However, mainly two limitations have been encountered in this study. First, the participants' sample in this study was taken from Punjab, Pakistan, and only consisted of secondary school science teachers. Pakistan consists of four provinces, so the findings cannot be generalized all over the country. Further study can be done to extend the study area to include more provinces and students as a sample. Secondly, the school's internal factors, such as laboratories, curriculum, and teacher quality have been used to assess students' science academic outcomes, while further study can be done on specific components within the broad categories, such as the impact of distinct teaching methods, detailed teacher qualifications, or specific curriculum content on science outcomes.

## 10 Conclusion and recommendations

The current study objectives were to determine school internal factors and their influence on students' science academic outcomes at the secondary school level. In this regard, three factors were examined, namely, science laboratories, curriculum, and teacher quality, with an eye on students' science academic outcomes. The data was analyzed using the Partial Least Squares (PLS-PM) Path Modeling technique. This approach assessed the influence and relationship between independent and dependent variables. The findings revealed that science laboratories, curriculum, and teacher quality positively influence students' science academic outcomes in science-related subjects. However, providing resources to establish well-equipped labs in schools is crucial. Hence, the Pakistani government should include hands-on experiments in science as an integral component of the formal evaluation process and promptly establish well-equipped scientific labs to enhance the efficacy of science education.

Additionally, it is recommended that the resourcefulness of scientific instructors be enhanced by supplying support material for science teaching and learning. It will enable students to engage in hands-on learning, cultivate critical thinking abilities, and explore innovative approaches. Meanwhile, conducting frequent reviews and edits of secondary textbooks to enhance their quality is essential. A limited selection of comprehensive scientific textbooks is now accessible in schools. Hence, those engaged in scientific education must develop comprehensive textbooks that possess the adaptability to align with the existing curriculum. Recruit educators with a degree in their respective subject area and complete professional accreditation. Motivating competent in-service instructors to enhance their quality and performance in schools is essential. Policymakers, teaching services authorities, school administrators, and professional bodies should cooperate to enhance teacher quality and capacity development by offering additional in-service training opportunities. It can be achieved through further studies and interactions with teachers from other institutions.

However, the study offers a novel contribution by examining the combined impact of laboratories, curriculum, and teacher quality on students' science academic outcomes at the secondary school level in Pakistan. Unlike prior studies that have often focused on these factors in isolation, this research integrates them to provide a comprehensive analysis using advanced statistical techniques. By focusing on an underexplored region, Punjab, Pakistan, the study adds valuable insights into how educational resources and teaching effectiveness influence science education in a developing country context, contributing to the broader discourse on improving science outcomes globally.

## Ethical statement

This study was approved by the Ethics Committee of Zhejiang Normal University, Jinhua, Zhejiang, China, with ethics approval number (ZSRT2024004). The research team strictly followed the ethical rules and principles established by the institution throughout the study to ensure all participants' responsible and respectful treatment.

## Funding statement

This research received no specific assistance from governmental, commercial, or non-profit entities.

## Data availability

All the relevant data are included in the manuscript and the supplementary document. No separate repository is attached.

## CRediT authorship contribution statement

**Hafiz Muhammad Ihsan Zafeer:** Writing – original draft, Methodology, Formal analysis, Data curation, Conceptualization. **Samra Maqbool:** Writing – review & editing, Software, Methodology, Formal analysis. **Yu Rong:** Writing – review & editing, Supervision, Resources, Funding acquisition. **Sufyan Maqbool:** Validation, Investigation, Data curation.

## Declaration of competing interest

The authors declare that they have no known competing financial interests or personal relationships that could have appeared to influence the work reported in this paper.
