# Supplementary material for: Mapping the relationship and influence of school internal factors with an eye towards students' science academic outcomes
Source: Heliyon. 2024 Sep 27;10(19):e38696. doi: 10.1016/j.heliyon.2024.e38696 (PMC11470565; doi:10.1016/j.heliyon.2024.e38696)
Supplement: Multimedia component 1 [file mmc1.docx]

**Appendix A: Questionnaire**

**Scale:** Strongly Disagree 1, Disagree 2, Neutral 3, Agree 4, Strongly Agree 5

**Scale:** Poor 1, Fair 2, Neutral 3, Very Good 4, Excellent 5

| **Name (optional)**  ***Gender**  ***Age** |  |
| --- | --- |
| ***Academic Qualification:** |  |
| ***Professional Training:** |  |
| ***Teaching Subjects:** |  |
| ***Experience:** |  |

|  | **Statements** |  |  |  |  |  |
| --- | --- | --- | --- | --- | --- | --- |
| **NO.** | **Laboratories** | **SD** | **D** | **N** | **A** | **SA** |
| 1 | My school has the facilities to accommodate the science students. |  |  |  |  |  |
| 2 | My school has the facility of science laboratories for students. |  |  |  |  |  |
| 3 | My school has enough apparatus/equipment to do the experiments in science laboratories. |  |  |  |  |  |
| 4 | My school has well knowing science teaching staff to assist experiments in lab. |  |  |  |  |  |
| 5 | In my school students do experiments in science lab on daily basis. |  |  |  |  |  |
|  | **Curriculum** |  |  |  |  |  |
| 1 | Science learning material (handbook & notes) is provided to students in my school. |  |  |  |  |  |
| 2 | Text books of science subjects contain sufficient material on scientific concepts and theories. |  |  |  |  |  |
| 3 | Sufficient material is available for learning science in my school. |  |  |  |  |  |
| 4 | Curriculum changes increase student motivation. |  |  |  |  |  |
| 5 | Curriculum positively affect classroom management. |  |  |  |  |  |
| 6 | As the curricula change, the quality of the learning environment increase. |  |  |  |  |  |
|  | **Teacher Quality** |  |  |  |  |  |
| 1 | Using charts, models and daily life examples are helpful to teach Science courses. |  |  |  |  |  |
| 2 | All teaching staff is good at explaining scientific things. |  |  |  |  |  |
| 3 | Cross questioning is an effective way during science lecture. |  |  |  |  |  |
| 4 | Teaching staff can easily lead students’ preparation for the science subjects. |  |  |  |  |  |
| 5 | The role of teachers in the school is a reflection of students' science outcomes. |  |  |  |  |  |
|  | **Academic Outcomes** | **P** | **F** | **N** | **VG** | **E** |
| 1 | How do you rate students’ academic outcomes? |  |  |  |  |  |
| 2 | How do you rate students’ science course’s scores? |  |  |  |  |  |
| 3 | How do you rate students’ Biology scores? |  |  |  |  |  |
| 4 | How do you rate students’ Chemistry scores? |  |  |  |  |  |
| 5 | How do you rate students’ Physics scores? |  |  |  |  |  |
